# Improving access and efficiency of ischemic stroke treatment across four Canadian provinces using a stepped wedge trial: Methodology

**DOI:** 10.3389/fstro.2022.1014480

**Published:** 2022-10-31

**Authors:** Noreen Kamal, Shadi Aljendi, Alix Carter, Elena A. Cora, Tania Chandler, Fraser Clift, Patrick T. Fok, Judah Goldstein, Gordon Gubitz, Michael D. Hill, Bijoy K. Menon, Brian Metcalfe, Kelly J. Mrklas, Stephen Phillips, Scott Theriault, Etienne Van Der Linde, David Volders, Heather Williams, ACTEAST Collaborators, Dylan Blacquiere

**Affiliations:** ^1^Department of Industrial Engineering, Faculty of Engineering, Dalhousie University, Halifax, NS, Canada; ^2^Division of Emergency Medical Services, Department of Emergency Medicine, Faculty of Medicine, Dalhousie University, Halifax, NS, Canada; ^3^Emergency Health Service Nova Scotia, Government of Nova Scotia, Halifax, NS, Canada; ^4^Department of Diagnostic Radiology, Faculty of Medicine, Dalhousie University, Halifax, NS, Canada; ^5^Halifax Infirmary, Queen Elizabeth II Health Sciences Centre, Halifax, NS, Canada; ^6^Division of Neurology, Department of Medicine, Faculty of Medicine, Dalhousie University, Halifax, NS, Canada; ^7^Saint John Hospital, Saint John, NB, Canada; ^8^Division of Neurology, Faculty of Medicine, Memorial University of Newfoundland, St. John's, NL, Canada; ^9^Health Sciences Centre, St. John's, NL, Canada; ^10^Department of Clinical Neurosciences, Cumming School of Medicine, University of Calgary, Calgary, AB, Canada; ^11^Department of Emergency Medicine, Faculty of Medicine, Memorial University of Newfoundland, St. John's, NL, Canada; ^12^Government of Newfoundland and Labrador, Emergency and Paramedicine Services Division, Department of Health and Community Services, St. John's, NL, Canada; ^13^Strategic Clinical Network, Alberta Health Services, Calgary, AB, Canada; ^14^Patient Partner, Halifax, NS, Canada; ^15^Dr. G. B. Cross Memorial Hospital, Clarenville, NL, Canada; ^16^Queen Elizabeth Hospital, Charlottetown, PE, Canada

**Keywords:** ischemic stroke, thrombolysis (for acute ischemic stroke), endovascular thrombectomy (EVT), stepped wedge trial design, *Improvement Collaborative*

## Abstract

**Introduction:**

Ischemic stroke is treatable with thrombolysis and/or endovascular treatment. Both treatments are highly time dependent, as faster treatment results in better outcomes. Utilization of both of these treatments is less than optimal, and treatment times continue to exceed the recommended benchmarks. An improvement intervention was launched across Atlantic Canada, which has four provinces: Nova Scotia (NS), New Brunswick (NB), Prince Edward Island (PEI), and Newfoundland and Labrador (NL). The intervention was conducted through the ACTEAST (Atlantic Canada Together Enhancing Acute Stroke Treatment) Project, which aimed to improve access and efficiency of treatment for acute ischemic stroke patients.

**Intervention and methods:**

The improvement intervention was a 6-month virtual *Improvement Collaborative* that consisted of each stroke center assembling an interdisciplinary team, 2 full-day Learning Sessions, five to six 1-h webinars, and a site visit for each team. The *Improvement Collaborative* intervention was implemented using a stepped-wedge trial design, where the intervention was delivered in 3 phases. The *Improvement Collaborative* was initially conducted with NS, followed by NB and PEI, and the final phase was with NL. The number of participants enrolled across all 34 hospitals were 98, 86, and 72 for NS, NB-PEI, and NL, respectively. The attendance at the Learning Sessions ranged from 43 to 81 across all 3 clusters. The attendance at webinars had a mean of 29.0 (SD 6.8), 26.0 (SD 6.3), and 19.0 (SD 8.5) for the NS, NB-PEI, and NL clusters respectively.

**(Anticipated) Results:**

We anticipate that an additional 3–5% of ischemic stroke patients will receive thrombolysis, EVT, or both. Additionally, we anticipate a reduction of 10–15 min in door-to-needle times across the region. This will translate to an increase in the proportion of ischemic stroke patients that will be discharged home from acute care.

**Discussion:**

High level of engagement is possible in an Improvement Collaborative Intervention when implemented using a stepped-wedge trial design. The highest level of engagement was observed in the NS cluster, which maybe because this province has the most established provincial stroke system. Physician engagement, a critical aspect of improvement, was high. COVID-19 restrictions likely led to lower attendance at site visits.

## Introduction

Revolutionary advances in ischemic stroke treatment have occurred over the past two decades from thrombolysis using IV (intravenous) alteplase in the mid-1990's (National Institute of Neurological Disorders and Stroke rt-PA Stroke Study Group, 1995; Wardlaw et al., 2009) to the recent ground-breaking trials showing the high efficacy of endovascular thrombectomy (EVT) in 2015 (Goyal et al., 2015, 2016). These treatments are synergistic. EVT is provided to a subset of ischemic stroke patients with the most severe form of ischemic stroke due to a large vessel occlusion, while alteplase is appropriate for a larger proportion of ischemic stroke patients. These treatments substantially reduce disability due to stroke and reduce healthcare costs.

Although alteplase and EVT are part of guideline care in Canada and around the world (Boulanger et al., 2018; Powers et al., 2018), less than optimal utilization rates for both of these treatments are observed. This evidence-to-practice gap is not a new phenomenon (Mallonee et al., 2006; Lang et al., 2007); however, the substantial economic and societal benefits of these therapies make it critical to pursue optimal uptake. Furthermore, the effectiveness of both of these treatments has been shown to be highly time dependent (Emberson et al., 2014; Saver et al., 2016). Significant reductions in onset to treatment time can be made by healthcare systems after the patient arrives in hospital and also after the 911 call is placed. Hospitals across North America (Fonarow et al., 2011) and around the world (SITS, 2021) have longer treatment times than the disease allows for. Therefore, there have been calls to reduce hospital alteplase treatment times to 30 min from hospital arrival (Kamal et al., 2014; Boulanger et al., 2018), and to create seamless transfer processes for more efficient access to EVT for patients living outside major urban centers (Kamal et al., 2017, 2018a). Reductions in arrival to treatment times have resulted in improved patient outcomes (Fonarow et al., 2014; Kamal et al., 2020). Additionally, studies found that reducing treatment times resulted in an increase in the proportion of patient that received treatment (Meretoja et al., 2012; Kamal et al., 2020).

**Figure 1:**
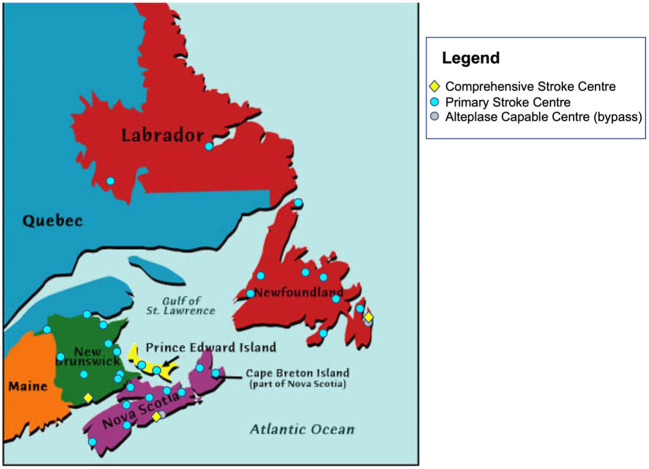
Location of all Primary Stroke Centers and Comprehensive Stroke Centers in Atlantic Canada. Also shown with a gray marker are two centers that are capable of alteplase treatment, but bypassed by ambulance due to proximity to another stroke center.

We aimed to improve both access and time efficiency to both alteplase and EVT treatment for acute ischemic stroke patients across Atlantic Canada through the ACTEAST (Atlantic Canada Together Enhancing Acute Stroke Treatment) project (Dalhousie University, 2020). Atlantic Canada is the eastern most region in Canada comprised of 4 provinces: Nova Scotia (NS), New Brunswick (NB), Prince Edward Island (PE), and Newfoundland and Labrador (NL). These provinces have small populations, and three of the provinces are smaller in geographic size; however, it should be noted that Newfoundland and Labrador is a geographically large province. The population and size of each province are provided in Table 1. Healthcare in Canada is a single-payer system that is administered provincially, which presents resource constraints for these small provinces. Each of these provinces has a well-established acute stroke system with designated stroke centers. Based on each system's prehospital protocols, paramedics transport suspected stroke patients to a designated stroke center. For the purposes of this paper and to ensure consistency, we will label all stroke hospitals capable of only alteplase treatment (thrombolysis) as Primary Stroke Centers (PSC) and hospitals capable of both alteplase and EVT treatment as Comprehensive Stroke Centers (CSC). Figure 1 shows all designated stroke hospitals across all 4 provinces, and Table 1 lists the number of each hospital type in each province.

**Table 1:** Population and geographic size of each of the Canadian Atlantic provinces.

**Province**	**Population (2022 estimate) (Statistic Canada, 2022)**	**Size (km^2^)**	**Number of PSCs**	**Number of CSCs**	**Number of alteplase capable centers (bypass)**
Nova Scotia	1,002,586	55,284	9	1	1
New Brunswick	797,102	72,907	9	1	0
Prince Edward Island	166,331	5,660	2	0	0
Newfoundland and Labrador	522,453	405,720	10	1*	1

PSC, Primary Stroke Center; CSC, Comprehensive Stroke Center; EVT, Endovascular Thrombectomy.

^*^The CSC in Newfoundland and Labrador began performing EVT treatment on June 20, 2022.

The intervention used in this study is a modified *Improvement Collaborative* that is described in detail in the next section. The trial design is a Stepped Wedge Trial, which is described in detail in Section Methods. Further details about how the *Improvement Collaborative* intervention is integrated into the Stepped Wedge Trial are also provided in Section Methods.

## Improvement Collaborative intervention

The intervention for this study is not a drug or device; the intervention is an improvement process that is used to make improvements across multiple hospitals. The Institute for Healthcare Improvement's (IHI) Breakthrough Series Collaborative model (referred here to as the *Improvement Collaborative*) (Institute for Healthcare Improvement, 2003) is used to implement improvement of acute stroke treatment across Atlantic Canada. This intervention has been used to improve and implement evidence-based best practice, though with mixed results (Flamm et al., 1998; Series and Kilo, 1998; Leape et al., 2000; Katzelnick et al., 2005; Prabhakaran et al., 2016; Kamal et al., 2020), and it has been utilized previously to reduce treatment times for ischemic stroke patients receiving alteplase (Langley et al., 2009; Sokovic et al., 2010).

The *Improvement Collaborative* is derived from the “Model for Improvement” utilizing the PDSA (Plan-Do-Study-Act) cycles, which was developed within the industrial engineering community (Shewhart, 1980; Deming, 1993). As described by the IHI, the *Improvement Collaborative* is a time-limited process typically 1-year long, and the process begins with sites formally enrolling a team to the *Improvement Collaborative*. It officially begins with the first Learning Session, which is typically a 2-day face-to-face workshop that includes presentations on the topic of improvement as well as details about the improvement process based on the Model for Improvement. During the Learning Session, there is a period allotted where each team plans their improvement activities. The Learning Session is followed by an Action Period. There are typically three Learning Sessions and Action Periods. Throughout the entire process, teams are supported with site visits, webinars, and data collection and feedback.

**Figure 2:**
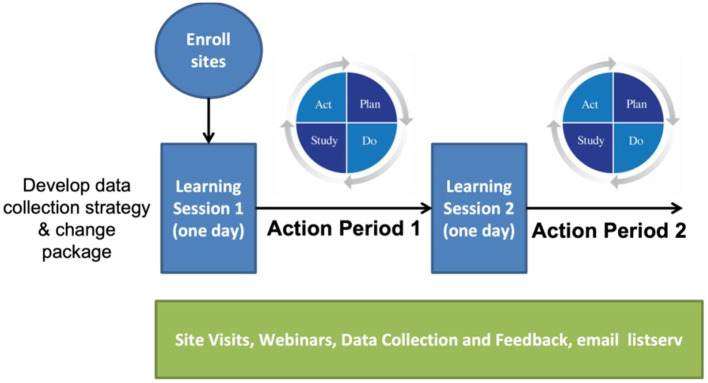
Improvement Collaborative process for ACTEAST project.

The *Improvement Collaborative* for the ACTEAST project was modified from the IHI model. The entire process was reduced in length from 1 year−6 months; there were two Learning Session/Action periods rather than three; and each Learning Session was 1-day long rather than 2 days in length. Figure 2 shows the modified *Improvement Collaborative* intervention. Additionally, all Learning Sessions and site visits were conducted virtually due to COVID-19 restrictions with the exception of one Learning Session for one cluster that used a hybrid delivery, which will be elaborated on in the next section.

## Methods

### Objectives

*The ACTEAST project aims to improve access and efficiency of stroke treatment across Atlantic Canada*. Specifically, ACTEAST will study the effectiveness of the proposed intervention through a rigorous quantitative study using a quasi-experimental stepped-wedge trial design. The primary objective for the ACTEAST project is to *increase the proportion of ischemic stroke patients that receive either thrombolysis or EVT by 5%*. A 5% increase was chosen because a previous study that utilized the *Improvement Collaborative* intervention resulted in an increase of 6.4% in thrombolysis rates, and current data suggests that around 9% of ischemic stroke patients received thrombolysis and 5% received EVT across Atlantic Canada.

The secondary objectives are as follows:

1. To reduce the median door-to-needle time (DNT) of all patients treated with alteplase or Tenecteplase
2. To increase the proportion of all ischemic stroke patients that are discharged home from acute care
3. To increase the proportion of all treated ischemic stroke patients that are discharged home from acute care
4. To reduce the hospital length of stay for all ischemic stroke patients
5. To reduce the hospital length of stay for all treated ischemic stroke patients
6. To reduce the door-in-door-out times for all patients transferred for EVT
7. To reduce the door-to-groin-puncture times for all EVT treated patients
8. To reduce the time to treatment from first medical contact (911 call).

The outcome measures are shown in numbers 2–5 in the list above. It should be noted the outcomes are being derived from administrative hospital data, as this is a pragmatic trial. Therefore, 90-day modified Rankin Score is not being collected, as it is not standard in clinical data collection across all stroke hospitals in these provinces.

### Stepped wedge trial

The *Improvement Collaborative* intervention described above was implemented within a stepped-wedge Trial to measure its effectiveness. In health and medicine, the randomized controlled trial design is widely accepted as the gold standard for evaluating the efficacy of an intervention on patient outcomes. However, in quality improvement and implementation interventions, it is not possible to randomize at the patient level since changes are made at the hospital level. For this reason, a cluster trial design is increasingly being proposed (Puffer et al., 2003), where an entire site or hospital is randomized to receive or not receive the intervention. However, there are several ethical issues with this design, as patients treated at hospitals in the control arm never receive the intervention. The stepped-wedge trial design alleviates these concerns by introducing the intervention to all participating hospitals in a sequential step-wise approach (Brown and Lilford, 2006).

**Figure 3:**
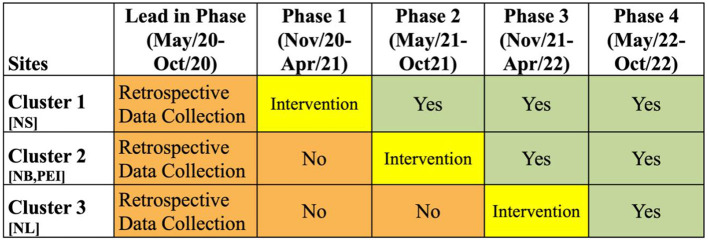
Stepped-Wedge Trial for ACTEAST Project. The orange periods show the time periods prior to the intervention. The green periods show the time periods after the intervention. The yellow shows the 6-month period for the *Improvement Collaborative* intervention. NS, Nova Scotia; NB, New Brunswick; PEI, Prince Edward Island; NL, Newfoundland and Labrador.

The evaluation of this *Improvement Collaborative* intervention will be conducted through a stepped-wedge trial (Figure 3). In this trial, all sites will be assigned to a group or cluster. Each cluster will go through the intervention at different times. Prior to going through the intervention, all clusters are in the control phase, while after the intervention, all clusters will have the intervention fully implemented. The intervention is 6 months in length, and it is the *Improvement Collaborative* described generally above. In Figure 3, the orange areas show the “control” periods where the intervention has not yet started, and the green areas show the periods after intervention has been completed. The data collected in the orange and the green phases will be analyzed, and data during the implementation phase will be excluded.

There are 3 clusters in this stepped-wedge trial, and the 6-month *Improvement Collaborative* intervention will be delivered in phases from November 2020 to April 2022. The first cluster will include all participating sites in the province of NS. Once this cluster ends, the second cluster will immediately begin, and it includes all participating sites in the provinces in NB and PE. The final cluster includes all participating sites in NL. Details of each intervention component for each of these clusters are described below. The ordering of these clusters were chosen based on the preference of health administrators in each of the provinces. Specifically, NS was chosen to be first as there was already good engagement of the stroke hospitals across the province, and NL wished to be last to allow them more time to organize and prepare for the intervention.

### Statistical analysis

Mixed-effects regression models (logistic or quantile as appropriate) that account for potential within-cluster and within hospital correlation of data will then be used to analyze the primary outcome and all secondary outcomes. Time will be included in these models as a continuous measure to account for any secular trends. The primary analysis will be a complete case analysis, with sensitivity analysis undertaken to include all participants under an appropriate model for missing data such as multiple imputation. All analysis will be adjusted for age, sex and baseline stroke severity.

### Ethics and trial registration

Ethical approval for this study was obtained from the NS Health Research Ethics Board (REB# 1025460), Health PEI Ethics Board, Horizon Health Network Research Ethics Board (ROMEO File #: 100906 and RS#: 2020-2893), Vitalité Health Network Ethics Office (ROMEO File Number: 101305), and Newfoundland and Labrador Health Research Ethics Board (Researcher Portal File #: 20210255 and Reference #: 2020.074).

The trial is registered with the ISRCTN registry (ISRCTN11109800, https://www.isrctn.com/ISRCTN11109800).

### Nova Scotia Improvement Collaborative intervention

The first cluster included all the stroke centers in NS. The NS *Improvement Collaborative* ran from November 2020 to April 2021. Site teams were recruited through the ACTEAST project co-investigators and collaborators. NS has a single health authority called Nova Scotia Health, and all hospitals fall under this authority. Leadership at all of the stroke centers were contacted and encouraged to participate in the ACTEAST *Improvement Collaborative*. Site formally enrolled by completing the enrolment form (see Supplemental material). The enrolment form included a *Call-to-Action*, information about the *Improvement Collaborative* (including expectations), and a form that asked for the list of team members. The suggested team members included an emergency physician, emergency nurse, paramedic, radiologist, diagnostic imaging representative, and neurologist (if the site had one). The enrolment form needed a signature from a physician and administrator. The team members made up the participants for the *Improvement Collaborative*.

For the NS cluster, 9 out of the 10 stroke centers enrolled teams for participation. A smaller primary stroke center located in a rural setting did not formally enroll although later during the course of the *Improvement Collaborative*, three individuals from the site did enroll. There were 2 additional teams that also enrolled. The thrombolysis-capable center located close to the CSC in NS that is bypassed by ambulance enrolled a team, as they see acute ischemic stroke patients that arrive by private vehicle. The other team that enrolled represented the provincial Emergency Health Services (EHS), which is the provincial ambulance service in NS. EHS is responsible for prehospital care and transport of patients, and they are also responsible for transfer between hospitals. The number of participants per site ranged from 4 to 15, and the mean was 8.6. The total number of participants was 98.

The NS *Improvement Collaborative* officially began with the first Learning Session. All participants were provided with a full *Change Package* that outlined the recommended changes to improve the treatment process (ACTEAST, 2020). They were also provided two publications authored by the PI: one that reviewed the literature on improving treatment times (Kamal et al., 2018b) and another that showed the results of a similar initiative in Alberta (Kamal et al., 2020). This session was held on November 9, 2020 from 9:00 a.m. to 4:00 p.m. in a hybrid fashion that included both in-person attendance and virtual attendance. Similarly, the presentations were also held both virtually and in-person. This session was approved for 5.25 Continuing Medical Education (CME) credits for both family physicians and specialists. The full agenda is provided in the Supplemental material. The day started with a stroke patient sharing their story, followed by an overview of the ACTEAST initiative. The NS stroke prehospital system and overall provincial stroke program were then presented. The evidence for alteplase treatment, EVT, and imaging for acute stroke were then presented. After the lunch break, a presentation on change ideas to improve their treatment process, and this was followed by a presentation from a NS hospital about the changes that they have implemented. The remainder of the afternoon was dedicated to each team planning their changes. There were provided planning worksheet that is shown in the Supplemental material. Each site reported their planned changes to the entire group. There were 81 attendees in total with 19 of them attending in-person.

The second Learning Session was held virtually on January 8, 2021 from 9:00 a.m. to 4:00 p.m. The agenda is provided in the Supplemental material. This session was approved for 5.0 h of CME credits. The day began with an overview of the provincial data to share performance toward the goals of the project. This was followed by a presentation from Lifeflight, the provincial air ambulance service, about arranging a fast transfer for EVT. There were a series of presentations from participating sites on their improvement efforts including the CSC in NS. The day ended with a similar team planning session using the same worksheet as Learning Session 1. Each team reported back their planned changes. There were 60 people that attended Learning Session 2.

There were six webinars that were held during the NS Improvement Collaborative. These sessions are summarized in Table 2. Each webinar had a presentation for around 40 min with 20 min for questions and discussion, and they were held using Zoom (Zoom Video Communications, San Jose, CA, USA). The attendance at each webinar ranged from 20 to 37 people. They were recorded, and the links to the recording were distributed to all participants.

**Table 2:** Webinars conducted during NS Improvement Collaborative.

**Date**	**Topic of Webinar**	**Number of attendees**
November 24, 2020	Evidence and best practice recommendations for alteplase and EVT treatment, and NIHSS	37
December 8, 2020	Case reviews of transfers for EVT	32
January 25, 2021	Skepticism around tPA and emergency consent	34
March 24, 2021	Changes at a NS Hospital (name supressed)	27
April 19, 2021	Changes at a NS Hospital (name supressed)	22
April 29, 2021	Endovascular thrombectomy	20

Virtual site visits were conducted with all teams *via* Zoom from February 19, 2021 to April 26, 2021. During the organization of the visits, participants were encouraged to invite others from their site. The visits included an overview of the ACTEAST project, an overview of suggested changes, and a presentation from the site on their improvements to date. Attendance at the site visits ranged from 4 to 20 people with a mean attendance of 8.8.

### New Brunswick/PEI Improvement Collaborative intervention

The next cluster included all stroke hospitals in NB and PE. The NB-PE *Improvement Collaborative* ran from May 2021 to October 2021; it began immediately after the NS *Improvement Collaborative* completed. New Brunswick has two health authorities: Horizon Health Network and Vitalité Health Network. Horizon serves the English communities and Vitalité serves the French communities, as NB is a fully bilingual province. PE has a single health authority called Health PEI.

The overall process for the NB-PE *Improvement Collaborative* was similar to the NS *Improvement Collaborative*. Most of the material was the same; however, all the documentation was available in both English and French. This included the presentations during Learning Sessions 1 and 2; however, all the presentations during the Learning Sessions and webinars were only given in English. Only processes and materials that differed from the NS *Improvement Collaborative* will be discussed in detail.

Site teams were recruited through ACTEAST's co-investigators and collaborators similar to the first cluster. Sites were formally enrolled using the ACTEAST enrolment form that was the same as the one used in the NS cluster. All 12 stroke centers enrolled in the ACTEAST Improvement Collaborative: 5 sites were from Horizon; 5 sites were from Vitalité; and 2 sites were from Health PEI. There were 2 smaller rural sites from Vitalité that enrolled as a single team, as they are close geographically and work closely together. One of the sites from Horizon is a CSC that provides EVT to the province of NB. The two sites in PE transfer their patients to the CSC in NS for EVT. The number of participants on each team ranged from 4 to 14, and the mean was 7.7. The total number of participants in the NB-PE *Improvement Collaborative* was 86.

The first Learning Session for the NB-PE *Improvement Collaborative* was held virtually on May 3, 2021 from 9:00 a.m. to 4:00 p.m. The same material was provided to participants: the *Change Package* and two publications authored by the PI (Section Ethics and trial registration). This session was approved for 5.25 h of CME credits for both family physicians and specialists. The agenda for this session is provided in the Supplemental material. The agenda followed the same topics as NS, but were contextually relevant to this cohort. A total of 73 people attended this session.

The second Learning Session for the NB-PE *Improvement Collaborative* was held virtually on June 25, 2021 from 9:00 a.m. to 4:00 p.m. The agenda is provided in the Supplemental material. This session was approved for 5.0 h of CME credits for both family physicians and specialists. The topics followed the same format as the second session for NS. There were 43 people that attended Learning Session 2.

There were five webinars that were held during the NB-PE *Improvement Collaborative*. These sessions are summarized in Table 3. The attendance at each webinar ranged from 20 to 35 people. They were recorded, and the links to the recording were distributed to all participants.

**Table 3:** Webinars conducted during NB-PE Improvement Collaborative.

**Date**	**Topic of Webinar**	**Number of attendees**
January 9, 2021	Consent for tPA	35
June 21, 2021	Code stroke protocol at a NS Hospital	25
September 29, 2021	Changes at a NB Hospital (name supressed)	28
October 19, 2021	Changes at a PE Hospital (name supressed)	20
October 31, 2021	Continue with improvement	20

Virtual site visits were conducted with all teams *via* Zoom from September 2, 2021 to October 26, 2021. The visits followed the same format as those for the NS *Improvement Collaborative*. Attendance at the site visits ranged from 3 to 12 people with a mean attendance of 7.0.

### Newfoundland and Labrador Improvement Collaborative intervention

The next cluster included all stroke hospitals in NL. The NL *Improvement Collaborative* ran from November 2021 to April 2022; it began immediately after the NB-PE *Improvement Collaborative* completed. NL has four health authorities: Eastern Health, Western Health, Central Health, and Labrador-Grenfell Health. The overall process for the NL *Improvement Collaborative* was similar to the previous 2 clusters. Most of the material was the same. Only processes and materials that differed from the NS *Improvement Collaborative* will be discussed in detail.

Site teams were recruited through ACTEAST's co-investigators and collaborators similar to the previous clusters. All 11 stroke centers and the thrombolysis-capable bypass hospital enrolled in the ACTEAST Improvement Collaborative. However, Central Health enrolled as a single team for both of their stroke centers, and Labrador-Grenfell Health enrolled as a single team for all 3 of their stroke centers. Eastern Health had 5 teams enroll; however, the bypass thrombolysis-capable hospital team later withdrew due to time constraints. Two teams enrolled from Western Health. The total number of teams was 9 from NL, and 8 after the one site withdrew. The number of participants on each team ranged from 5 to 14, and the mean was 7.9. The total number of participants in the NL *Improvement Collaborative* was 72.

The first Learning Session for the NL *Improvement Collaborative* was held virtually on November 26, 2021 from 9:00 a.m. to 4:00 p.m. This session was postponed for 2 weeks due to an event that severely impacted all their electronic systems. The same material was provided to participants: the *Change Package* and two publications authored by the PI (Section Ethics and trial registration). This session was approved for 5.0 h of CME credits for both family physicians and specialists. The agenda for this session is provided in the Supplemental material. The agenda followed the same topics as the previous clusters, but were contextually relevant to this cohort. A total of 46 people attended this session.

The second Learning Session for the NL *Improvement Collaborative* was held virtually on February 4, 2022 from 9:00 a.m. to 4:00 p.m. This session was postponed by 3 weeks due to the negative staffing impact that the Omicron variant was having across all NL hospitals. The agenda is provided in the Supplemental material. This session was approved for 4.75 h of CME credits for family physicians and 4.25 h of CME credits for specialists. The topics followed the same format as the second session for NS. There were 50 people that attended Learning Session 2.

There were five webinars that were held during the NL Improvement Collaborative. These sessions are summarized in Table 4. The attendance at each webinar ranged from 12 to 34 people. They were recorded, and the links to the recording were distributed to all participants.

**Table 4:** Webinars conducted during NL Improvement Collaborative.

**Date**	**Topic of Webinar**	**Number of attendees**
February 22, 2022	Tenecteplase and the future of stroke thrombolysis	34
March 10, 2022	PEI Hospital (name supressed) changes to reduce DTN	16
March 31, 2022	Future of stroke treatment and neuroprotectants	17
April 13, 2022	Improvements at a NS Hospital (name supressed)	12
April 14, 2022	Improvements at a NL Hospital (name supressed)	18

Virtual site visits were conducted with all teams *via* Zoom from April 1 to 29 2022. The visits followed the same format as those for the previous clusters. Attendance at the site visits ranged from 3 to 16 people with a mean of 8.0.

### Summary of all Improvement Collaboratives

**Figure 4:**
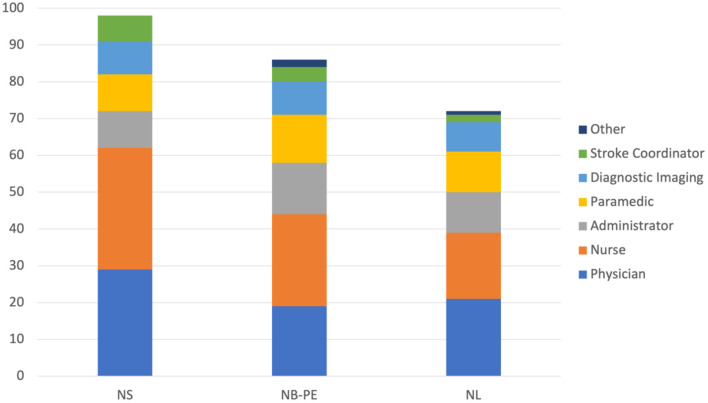
Number of participants from each professional groups for each cluster. NS, Nova Scotia; NB, New Brunswick; PE, Prince Edward Island; NL, Newfoundland and Labrador.

A summary of the engagement across all 3 clusters for each component of the *Improvement Collaborative* is provided in Table 5. The level of engagement across all clusters was similar with the greatest engagement occurring in NS. Figure 4 shows the distribution of professions for all participants in each cluster. Physicians and nurses made up the majority of participants' professions with paramedics, diagnostic imaging professionals, and administrators also contributing to a large portion of the participants. The distribution was similar across all 3 clusters for each professional group.

**Table 5:** Summary of engagement across all 3 clusters for each component of the Improvement Collaborative.

**Component**	**NS**	**NB-PE**	**NL**
Percent of stroke centers participating*	91%	100%	100%**
Total number of participants	98	86	72
Total number of teams	11	12	9
Mean number of participants per team (SD)	8.6 (3.17)	7.7 (3.00)	7.9 (2.85)
Attendance at learning session 1	81	73	46
Attendance at learning session 2	60	43	50
Number of webinars	6	5	5
Mean attendance at webinars (SD)	29.0 (6.8)	26.0 (6.3)	19.0 (8.5)
Mean attendance at site visits (SD)	8.8 (4.5)	7.0 (2.8)	8.3 (5.7)

^*^Including tPA capable bypass centers.

^**^One team withdrew halfway through the Improvement Collaborative.

## (Anticipated) Results

The key improvements that will be made across Atlantic Canada will be related to the process measures for acute ischemic stroke treatment. We anticipate that there will be an increase in the utilization of thrombolysis. Primarily, ischemic stroke patients are treated with alteplase (tissue plasminogen activator, tPA) across the region; however, two sites, one in NS and the other in PE, participated in the ACT (Alteplase compared to Tenecteplase) trial (Menon et al., 2022); therefore, some patients were treated with Tenecteplase (TNK) in this region. Furthermore, the trial successfully ended at the end of 2021, and the results were published in the first half of 2022; therefore, some sites may have transitioned to TNK after the results were published in June 2022. In addition to thrombolysis, an increase in acute ischemic stroke patients that received EVT is anticipated. This will be particularly apparent for patients from PSCs that are transferred to CSCs for EVT. The increase in the number of patients treated with EVT will be notable in NL, as they began offering EVT in June 2022. We anticipate that 3–5% additional ischemic stroke patients will receive reperfusion therapies with thrombolysis, EVT, or both.

The second area of key anticipated improvements in the processes will be in reduction of door-to-needle time (DNT). This is the time from hospital arrival to the start of thrombolysis. We anticipate a reduction of 10–15 min in DNT across the region.

The translation of these results to patient outcomes will be assessed using administrative data. The primary outcome measure is proportion of patients discharged home from acute care for both all ischemic stroke patients and patients that receive either alteplase or EVT treatment. It is anticipated that both groups will see an increase of 3–5% in patients discharged home from acute care.

## Discussion

The level of engagement in the 6-month *Improvement Collaborative* that was observed for each cluster was high. There was engagement from all stroke centers with active improvement efforts being conducted by all teams. Engagement was the highest in NS, which is likely because they have the most established stroke system with a stroke coordinator assigned to each stroke center. The role of the stroke coordinator played a significant part in engaging their site and managing improvements. It should be noted that PE does have an active stroke coordinator and a well-established provincial stroke program; however, due to the small size of this province, their engagement results were combined with NB.

Physician engagement has been established as being critical for improving health systems (Marsden et al., 2012; Kaissi, 2014). The ACTEAST Project was able to achieve a good level of physician engagement across all 3 clusters with 29.6, 22.1, and 29.2% of all participants being physicians in NS, NB-PE, and NL, respectively. The key efforts that were made to engage physicians were having CME credits for the Learning Sessions and encouraging each site to have a physician representative on their team. This is a high level of engagement, as teams were encouraged to have representation from 6 different professions, and physician made up approximately a quarter of all participants. Furthermore, they were either the largest or second largest professional group in each cluster.

Participation from the other professional groups were similar across all 3 clusters. However, there were more administrators in the NB-PE and NL clusters (16.3 and 15.3%, respectively) compared to the NS cluster (10.2%). Similarly, representation from the prehospital area (shown as paramedics) was greater in NB-PE and NL clusters (15.1 and 15.3%, respectively) compared to the NS cluster (10.2%). The larger number of administrators in the NB-PE and NL clusters may be because they have less established stroke system, so administrators were participating to ensure the necessary supports were provided to sites.

The components for the *Improvement Collaborative* across all 3 clusters were delivered virtually due to gathering restrictions caused by the COVID-19 pandemic. This likely had the greatest effect on attendance at the virtual site visits, which only saw mean attendance of 7.0–8.8 participants per cluster. This is much lower than previous *Improvement Collaborative* that was conducted in the province of Alberta, where the site visits were done in-person and often conducted as a dinner meeting, which saw a mean attendance of 28 people (Kamal et al., 2019). Therefore, the site visits were likely less impactful in ACTEAST. Additionally, this shows the importance of having face-to-face site visits with an added incentive to attend such as a dinner.

## Data availability statement

The original contributions presented in the study are included in the article/Supplementary material, further inquiries can be directed to the corresponding author.

## Ethics statement

Ethical approval for this study was obtained from the NS Health Research Ethics Board (REB# 1025460), Health PEI Ethics Board, Horizon Health Network Research Ethics Board (ROMEO File #: 100906 and RS#: 2020-2893), Vitalité Health Network Ethics Office (ROMEO File Number: 101305), and Newfoundland and Labrador Health Research Ethics Board (Researcher Portal File #: 20210255 and Reference #: 2020.074).

## ACTEAST collaborators

Dylan Blacquiere, John Blake, Ashley Boyce, Greg Browne, Cassie Chisholm, Jamie Drapeau, Rachel Goss, Edgar Goulette, Trish Helm-Neima, Carolyn MacPhail, Alier Marrero, Matthew Murphy, Susannah Piercey, Amer Qureshi, Julie Savoie, Nicole Tupper, Wendy Simpkin, Shauna Sommerville, and Peter Vanberkel.

## Author contributions

NK conceived the study, wrote the manuscript, and carried out the intervention. AC, EC, TC, PF, JG, MH, BKM, BM, KM, SP, ST, EV, DV, and HW provided input into the study design and proposal. SA, AC, EC, TC, PF, JG, GG, MH, BKM, BM, SP, ST, EV, DV, and HW assisted in carrying the intervention. All authors provided feedback on the manuscript. All authors contributed to the article and approved the submitted version.

## Funding

This study was funded by the Canadian Institutes of Health Research (CIHR) Project Grant (PJT-169124).

## Conflict of interest

Author NK is part equity owner of DESTINE Health Inc.

The remaining authors declare that the research was conducted in the absence of any commercial or financial relationships that could be construed as a potential conflict of interest.

## Publisher's note

All claims expressed in this article are solely those of the authors and do not necessarily represent those of their affiliated organizations, or those of the publisher, the editors and the reviewers. Any product that may be evaluated in this article, or claim that may be made by its manufacturer, is not guaranteed or endorsed by the publisher.
