# Supplementary material for: Improving access and efficiency of ischemic stroke treatment across four Canadian provinces using a stepped wedge trial: Methodology
Source: Front Stroke. 2022 Oct 31;1:1014480. doi: 10.3389/fstro.2022.1014480 (PMC12802605; doi:10.3389/fstro.2022.1014480)
Supplement: Supplementary file 1 [file Data_Sheet_1.docx]

**ACTEAST Methodology: Supplemental**

1. **Enrolment Form (3 pages)**

**
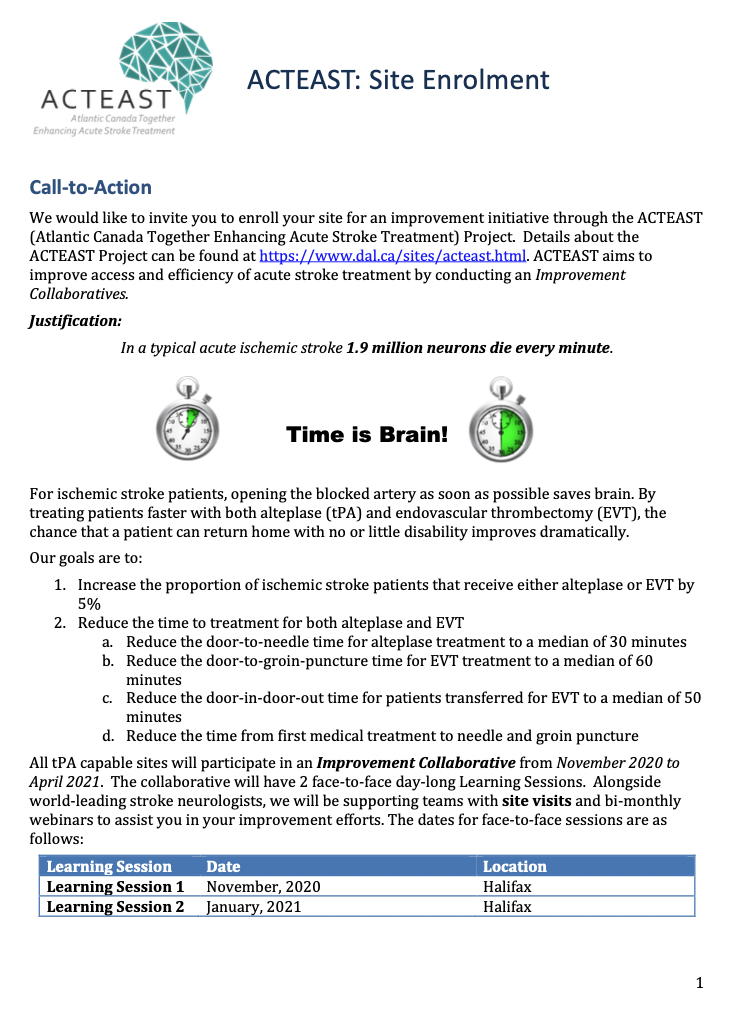
**

**
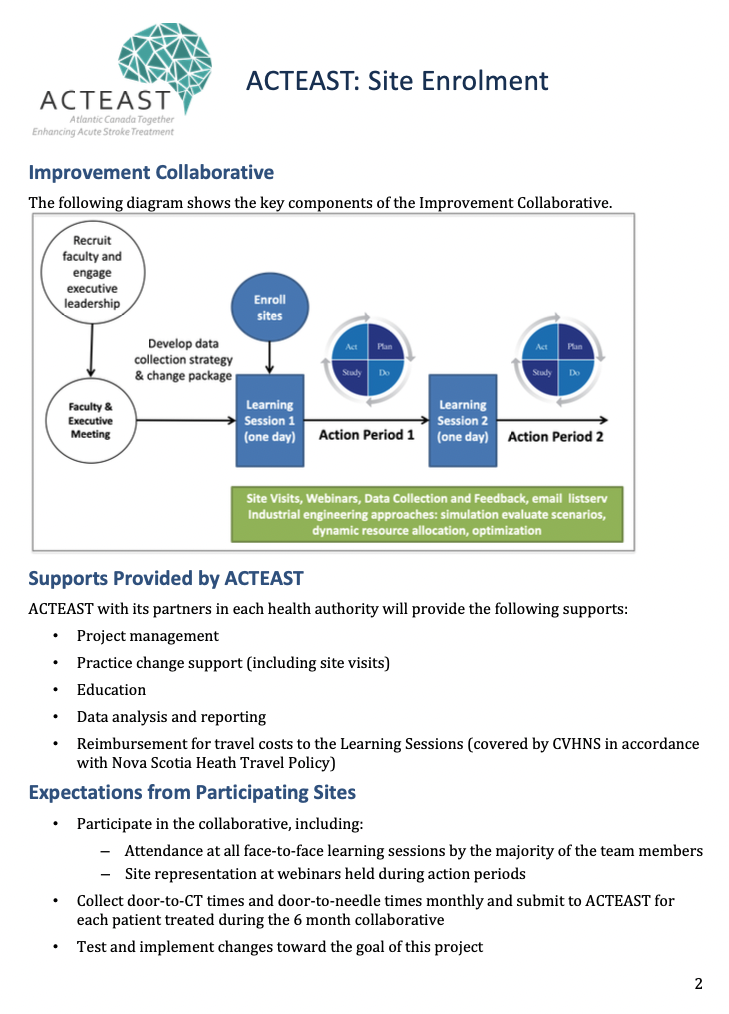
**

**
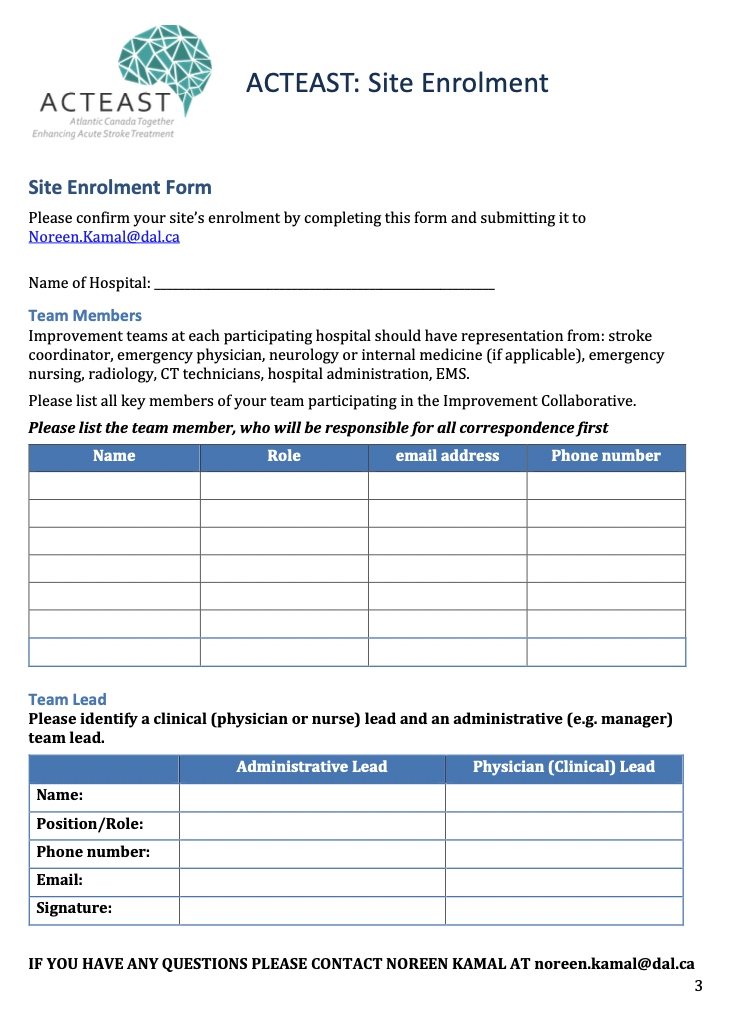
**

1. **Nova Scotia Improvement Collaborative – Learning Session 1 Agenda**

**
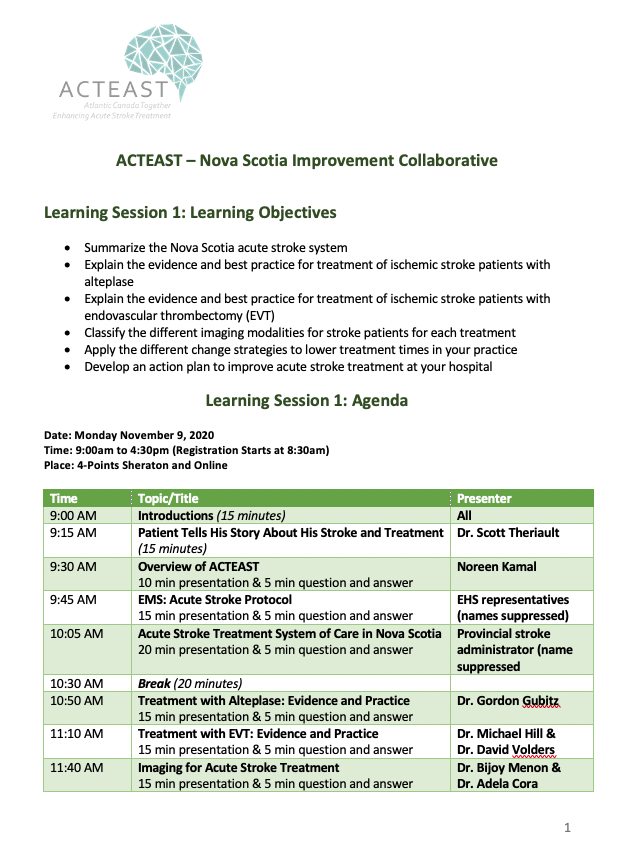
**

**
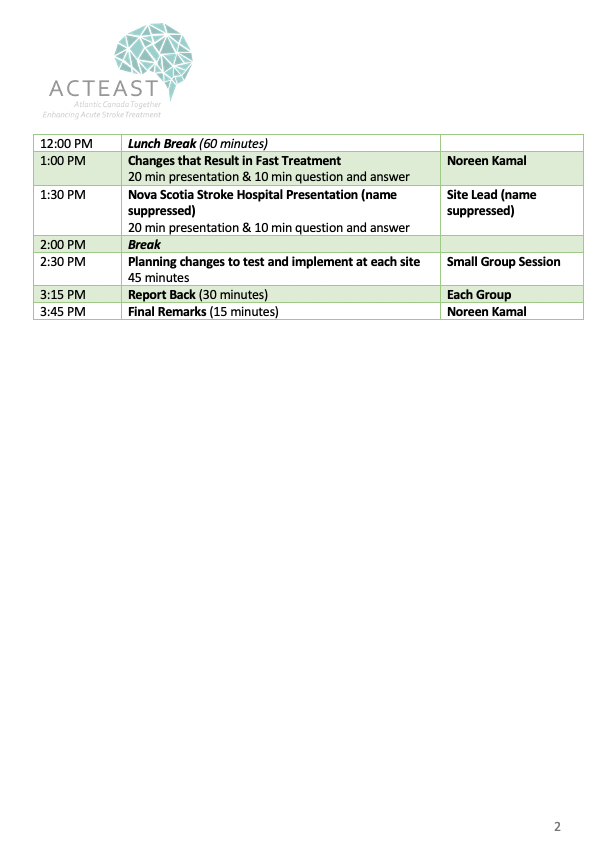
**

1. **Nova Scotia Improvement Collaborative – Action Planning Sheet**

**
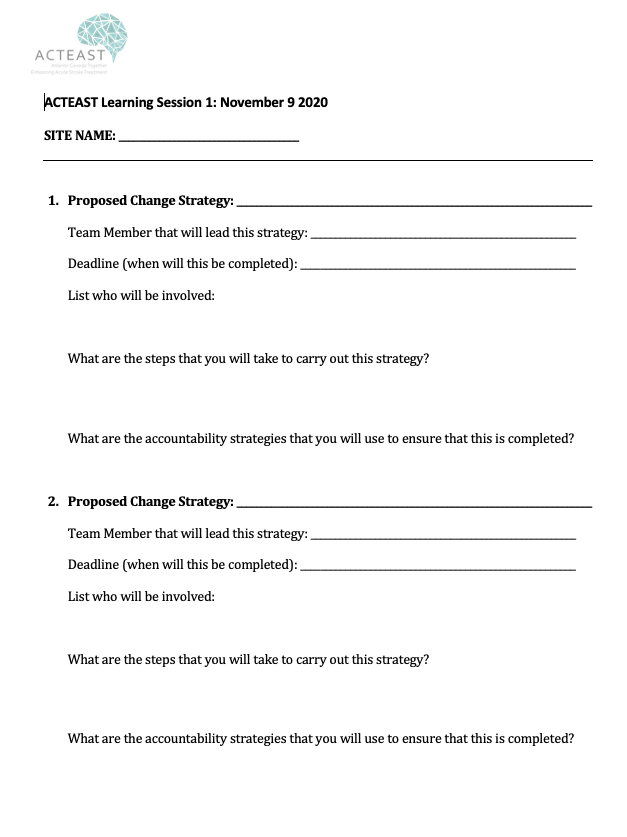
**

**
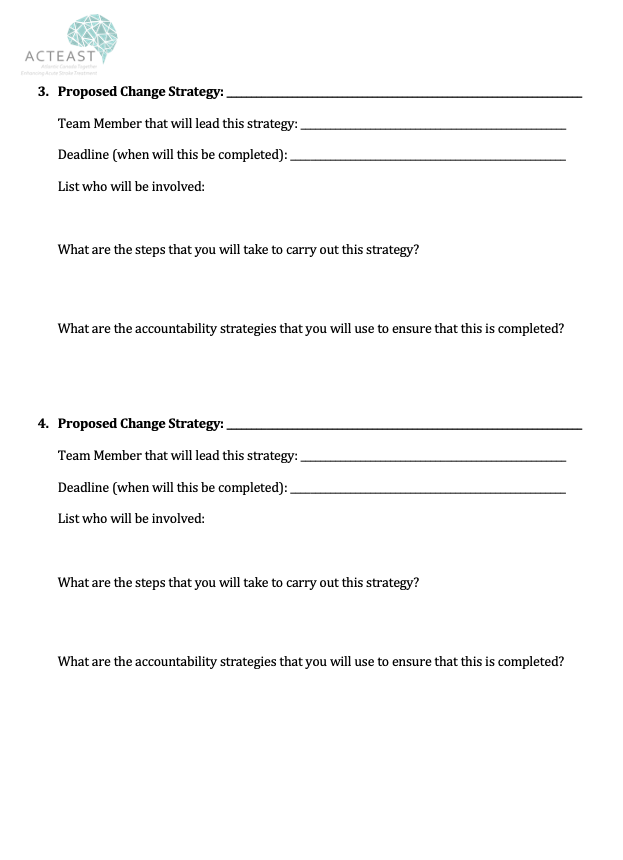
**

1. **Nova Scotia Improvement Collaborative – Learning Session 2 Agenda**

**
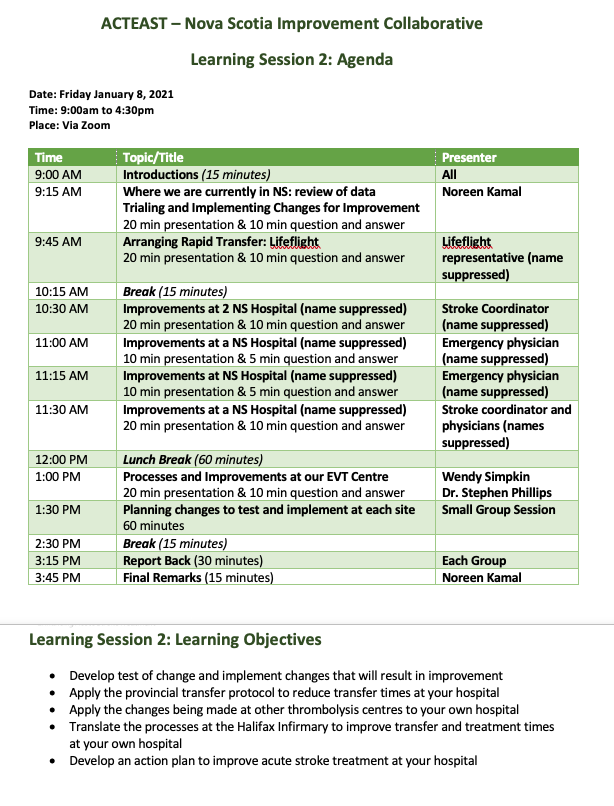
’**

1. **NB-PEI Improvement Collaborative – Learning Session 1 Agenda**

**
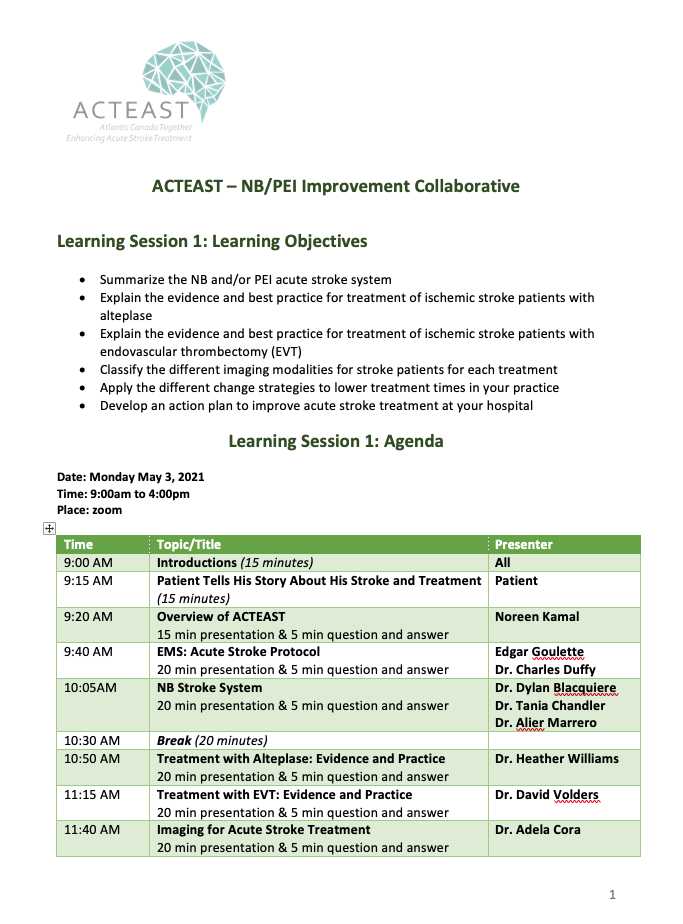
**

**
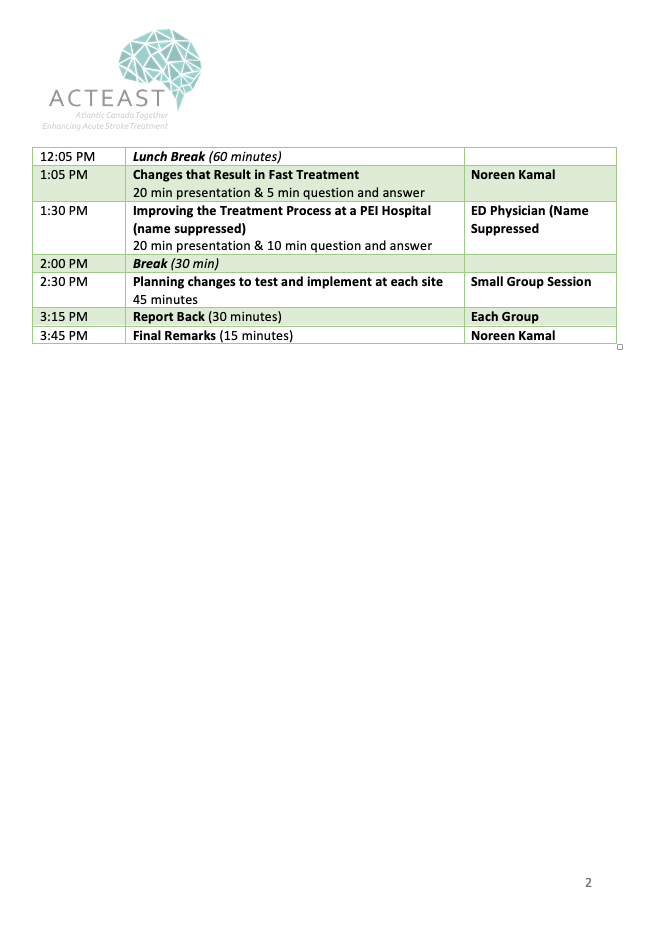
**

1. **NB-PEI Improvement Collaborative – Learning Session 2 Agenda**

**
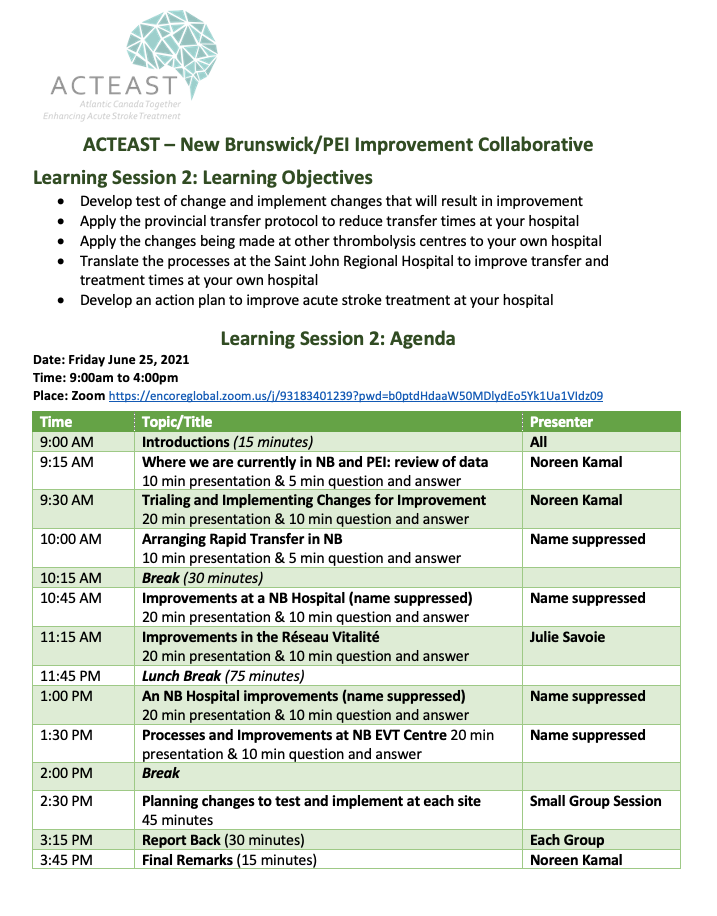
**

1. **NL Improvement Collaborative – Learning Session 1 Agenda**

**
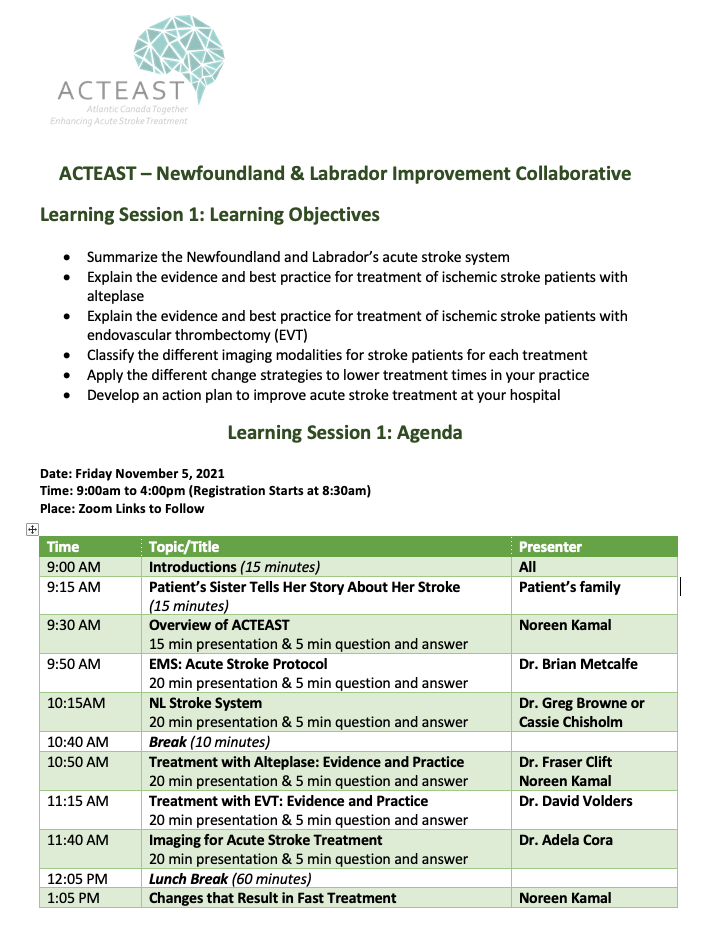
**

**
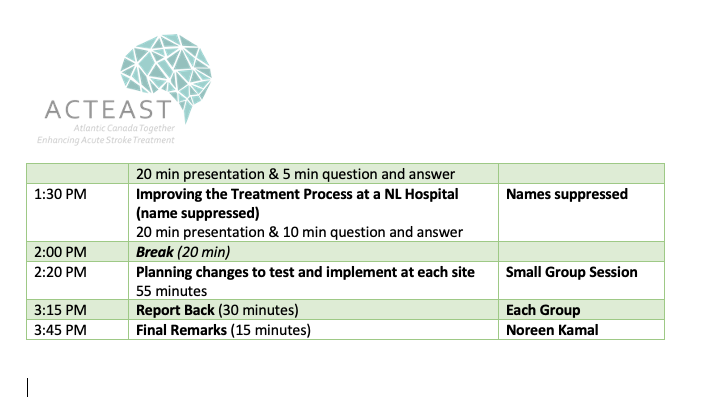
**

1. **NL Improvement Collaborative – Learning Session 2 Agenda**

**
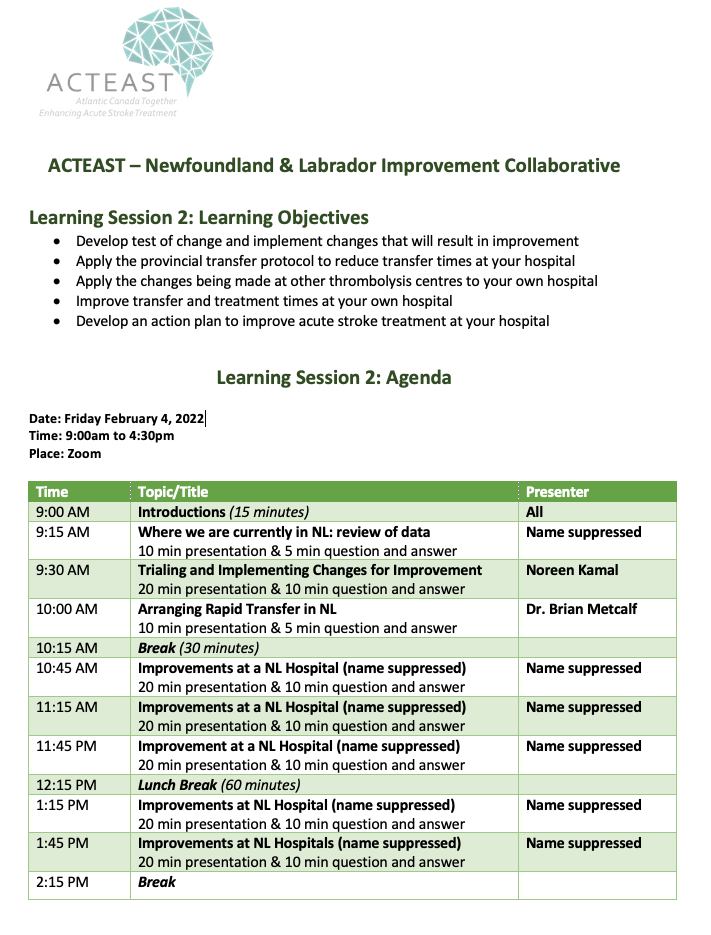
**

**
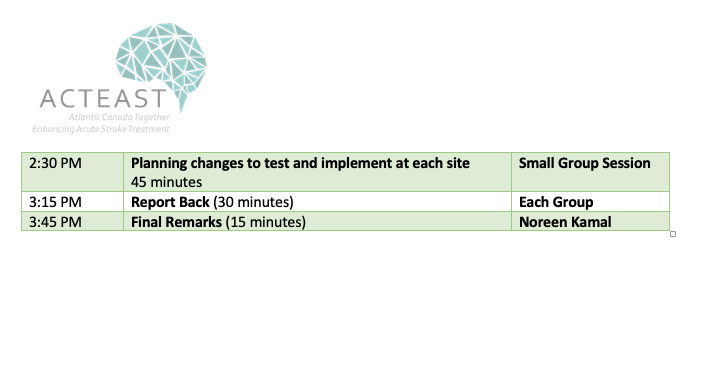
**
